# Clinical characteristics, management, and 5-year survival compared between no standard modifiable risk factor (SMuRFless) and ≥ 1 SMuRF ACS cases: an analysis of 15,051 cases from Pakistan

**DOI:** 10.1186/s12872-023-03355-z

**Published:** 2023-06-24

**Authors:** Sana Sheikh, Ghazal Peerwani, Bashir Hanif, Salim Virani

**Affiliations:** 1 Department of clinical research cardiology, Tabba Heart Institute, ST-1, Federal ‘B’ area, block 2, Karachi, 75950 Pakistan; 2 Department of clinical cardiology, Tabba Heart Institute, Karachi, Pakistan; 3 The Aga Khan University, Karachi, Pakistan

**Keywords:** Acute coronary syndrome, Immediate mortality, Long-term mortality, Low middle-income country, South Asia, Standard modifiable risk factors

## Abstract

**Background:**

There has been an increase in Acute Coronary Syndrome (ACS) patients without standard modifiable risk factors i.e. hypertension, diabetes, dyslipidemia, and tobacco use (SMuRFless) compared to the patients with ≥ 1 SMuRF but this has not been studied in South Asia despite them being a high-risk population. We conducted a comparative analysis of first episodes of ACS cases admitted to a tertiary cardiac center in Pakistan between SMuRFless and ≥ 1 SMuRF patients for clinical presentation, management, in-hospital, and 5-year mortality.

**Methods:**

We undertook a retrospective study and data of 15,051 patients admitted at Tabba Heart Institute (THI) with the first episode of ACS was extracted from Chest Pain-MI™, and the CathPCI Registry® registry affiliated with the National Cardiovascular Data Registry (NCDR®), USA. Logistic regression and Cox proportional algorithm yielded odds ratio (OR) and hazard ratios (HR) with 95% confidence interval (CI) for associated factors of in-patient and 5-year mortality.

**Results:**

There were 15% SMuRFless cases and in-hospital mortality was 4.1% in SMuRFless vs. 3.9% in the ≥ 1 SMuRF group (p-0.59), the difference remained insignificant after adjusting for age, gender, Killip class, multivessel disease, type of ACS, percutaneous coronary intervention (PCI) and coronary artery bypass grafting (CABG) (Adjusted OR:1.1 [0.8, 1.3]. Unadjusted 5-year mortality was 40% lower in the SMuRFless group but the difference was insignificant after adjusting for age, gender, disease at presentation, its severity, and management (Adjusted HR 0.7 95% CI[0.5, 1.0]). STEMI, NSTEMI, Killip class, and multivessel disease increased the risk of overall 5-year mortality.

**Conclusion:**

In-hospital and 5-year mortality was not different between the SMuRFless and ≥ 1 SMuRF group, there is a need to understand mediators of immediate and long-term mortality risk in SMuRFless patients.

## Background

There is an increase in coronary heart diseases (CHD) in lower-middle-income countries (LMIC) with an increase in standard modifiable risk factors (SMuRF) such as diabetes mellitus, hypertension, dyslipidemia, and tobacco use [1]. South Asia (SA) is a lower-middle-income region and SAs are known to have an early presentation (average 10 years earlier than other ethnicities) and higher rates of ST-elevation myocardial infarction (STEMI) despite a lower number of SMuRFs or presenting without any SMuRF (termed as SMuRFless) [2, 3]. The literature on SMuRFless ACS cases is limited to developed countries. Vernon et al. analyzed Australian national data and found the increasing trend of SMuRFless STEMI patients over approximately two decades (14%-1999; 23%-2017) [4]. When it comes to outcomes, findings from the Swedish registry found an increased rate of in-hospital, 30-day, and long-term mortality in SMuRFless compared to SMuRFs group [5]. So far the only study from the Asian region is from the high-income country of Singapore which reported higher short-term and long-term mortality in the SMuRFless ACS group [6]. Considering the higher probability of developing atherosclerosis among SAs and having unfavorable outcomes [7], there is a need to investigate SMuRFless ACS cases in this population.

Pakistan is a south Asian country grappling with the double burden of diseases and the health care resources are mainly invested in communicable diseases and maternal and child health [8]. The National Health Vision 2016–2025 document has no mention of cardiovascular disease despite cardiovascular diseases being the leading cause of adult mortality [9]The data on cardiovascular diseases in general, and on burden of disease in risk free individuals is needed to highlight the severity of the problem. Hence, we aimed to estimate the rate of SMuRFless ACS cases, and compare their hospital management, in-hospital mortality, post-procedure complications, and 5-year survival compared with ACS patients with ≥ 1 SMuRF presenting to a tertiary care cardiac center in Pakistan.

## Methods

A retrospective study on patients with no prior history of ACS, admitted to Tabba Heart Institute (THI) for the first episode of ACS from July 2013-June 2021was conducted. The data was extracted from Chest Pain-MI™ and CathPCI Registry®, National Cardiovascular Data Registry (NCDR®) of THI which is a non-profit, tertiary-level cardiac hospital in Karachi, Pakistan. It serves as one of the key referral sites for primary PCI in the city and on average 4000 ACS cases are admitted per year. The hospital is the only cardiac center in Pakistan affiliated with NCDR® since 2013 and has been the recipient of the “Platinum Performance Achievement Award” for the Chest Pain-MI™ registry in 2022. The hospital maintains an electronic medical record (EMR) system for all patients receiving emergency, outpatient, and inpatient services. Data of all the patients admitted with the diagnosis of ACS and those who undergo cath and PCI were extracted from the EMR and by interviewing patients. The data is then entered in Chest Pain-MI™ and CathPCI Registry®. Data is submitted and benchmarked quarterly by NCDR®.

For this study, data of all the patients with the first episode of ACS admitted to the study hospital from 2012 to 2021 was extracted. Patients with a history of heart failure, myocardial infarction, prior PCI or CABG, and stroke were excluded. Moreover, any subsequent admissions were excluded from the analysis. After applying the eligibility criteria to the dataset of 23,974 patients presenting with ACS, 15,051 ACS cases were included in the analysis.

The selected cohort was then categorized into SMuRFless and ≥ 1 SMuRF based on the absence or presence of hypertension, diabetes mellitus, dyslipidemia, and tobacco use documented in the patients’ EMR, and by interviewing the patients. Hypertension, diabetes mellitus, and dyslipidemia were defined as being diagnosed by a physician at any time, and diet and lifestyle modifications advised by the physician to control the disease or taking antihypertensive/antidiabetic medications, or lipid-lowering agents at home. Tobacco use was defined as currently smoking tobacco in any form or taking smokeless tobacco.

Study outcomes were mortality during the hospital stay for the ACS i.e. in-hospital death, and mortality at 5 years after the index episode. Other outcomes included complications that occurred during the hospital stay. These complications included reinfarction during the hospital stay, development of cardiogenic shock, heart failure, stroke, acute kidney injury requiring dialysis, major bleeding (bleeding requiring medical intervention or a drop of 3 gms of hemoglobin), and stent thrombosis. The complications were defined as per NCDR® Chest Pain-MI™ and CathPCI Registry® in the United States [10].

### Statistical analysis

The mean and standard deviation for continuous variables and the frequency and percentage for categorical variables were calculated. The characteristics and outcomes of SMuRFless ACS cases were compared with ≥ 1 SMuRF ACS cases using chi-square and independent student’s t-test. When assumptions of normality were violated or data was sparse, alternate non-parametric tests like Mann-Whitney, fisher exact, or linear by linear association were used to compare the groups. We then performed multivariable logistic regression to determine factors associated with in-hospital mortality, and crude and adjusted odds ratio (AOR) with 95% confidence interval (CI) were estimated. The in-hospital mortality model was adjusted for age, gender, type of ACS, Killip class, number of diseased vessels, undergoing PCI, and CABG. The Kaplan-Meier survival curves were made and the log-rank test was applied to compare the unadjusted 5-year mortality between the groups. Cox proportional algorithm was used to determine factors of 5-year mortality and unadjusted and adjusted hazard ratios (HR) with 95% CI were calculated. The model was adjusted for age, gender, type of ACS, Killip class, number of diseased vessels, undergoing percutaneous coronary intervention (PCI), and medicines prescribed at discharge.

## Results

**Fig. 1:**
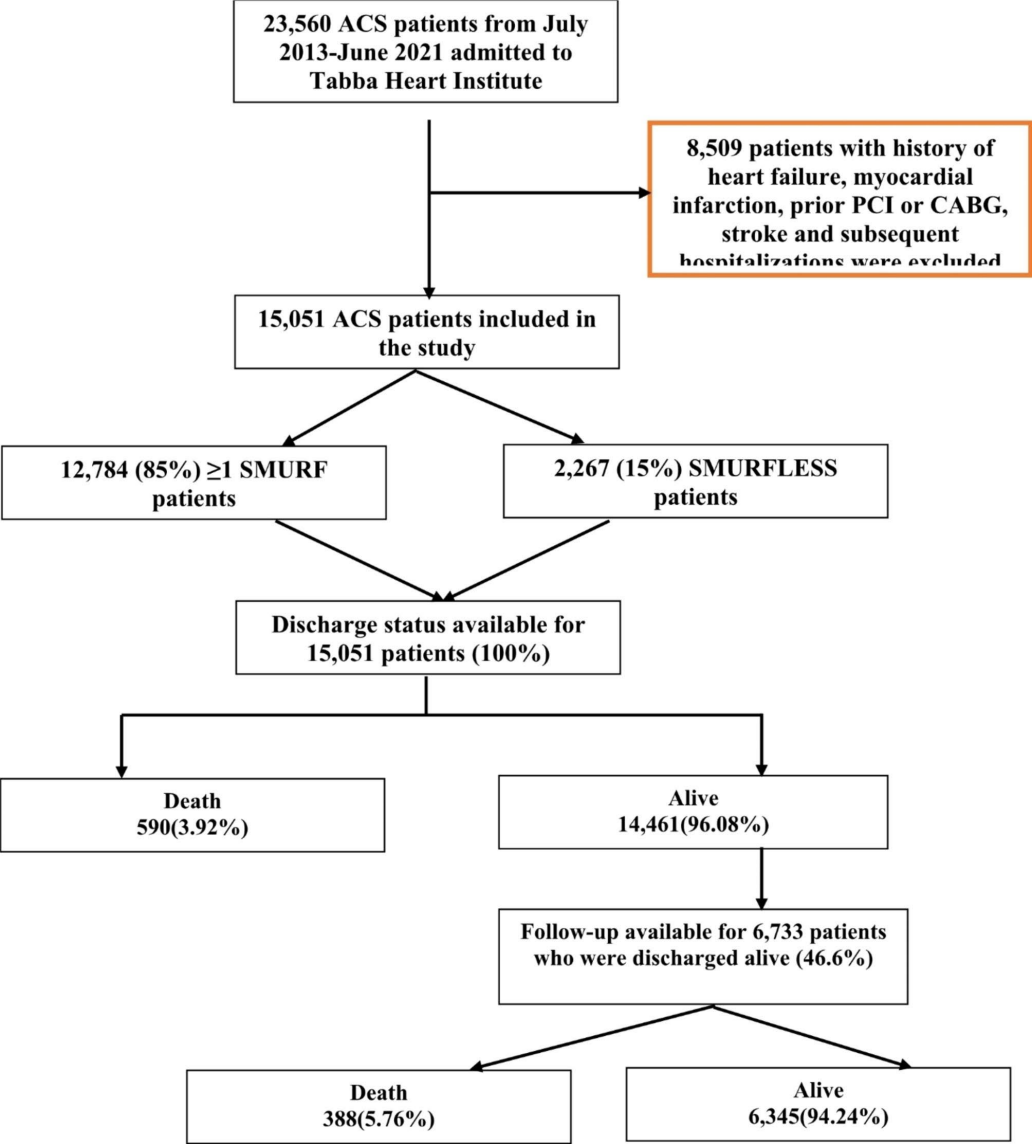
Flowchart of study participants

There were 2267/15,051 (15.0%) of the ACS cases without any SMuRF. Flowchart of participants and their followup is given in Fig. 1. Table: 1 displays the baseline characteristics of the participants. SMuRFless patients were on average slightly younger and had a higher proportion of men (p-value < 0.01).

**Table 1:** Baseline characteristics of the participants

Variables	Total	**SMuRFless**	≥ 1 SMuRF	p-value
	**N = 15051**	**N = 2267**	N = 12784	
	n (%)	n (%)	n (%)	
Age (mean years)	57.6 ± 11.5	56.2 ± 13.0	57.8 ± 11.2	< 0.01
Women	3626 (24.1%)	436 (19.2%)	3190 (25.0%)	< 0.01
Welfare received for the episode of care	556 (3.7%)	91 (4.0%)	465 (3.6%)	0.38
Family history of premature CAD	3234 (21.5%)	481 (21.2%)	2753 (21.2%)	0.72
Peripheral arterial disease	35 (0.2%)	4 (0.2%)	31 (0.2%)	0.52
Prior valve surgery*	32 (0.2%)	10 (0.4%)	22 (0.2%)	0.02
History of cancer	66 (0.6%)	9 (0.6%)	57 (0.6%)	0.87
Dyslipidemia	3008 (20.0%)	0 (0%)	3008 (23.5%)	-
Hypertension	8784 (58.6%)	0 (0%)	8784 (68.7%)	-
Diabetes mellitus	6659 (44.2%)	0 (0%)	6659 (52.3%)	
Tobacco use ever	4315 (28.7%)	0 (0%)	4315 (33.8%)	-
Smokeless tobacco use	421 (7.4%)	0 (0%)	421 (8.1%)	-
**Number of cigarettes**				-
< 10/day	528 (42.1%)	0 (0%)	528 (42.1%)	
> 10/day	727 (57.9%)	0 (0%)	727 (57.9%)	
**Number of SMuRFs**				-
0	2267 (15.1%)	2267 (100%)	0 (0%)	
1	5433 (36.1%)	0 (0%)	5433 (42.5%)	
2	5005 (33.3%)	0 (0%)	5005 (39.2%)	
3	2061 (13.7%)	0 (0%)	2061 (16.1%)	
4	285 (1.9%)	0 (0%)	285 (2.2%)	

*Fisher exact test was applied

*CAD = Coronary artery disease*

*SMuRF = Standard Modifiable Risk Factor*

The clinical presentation and management data are presented in Table: 2. More patients with cardiac arrhythmia and STEMI were in the SMuRFless group (p-value < 0.01). A higher proportion of NSTEMI, multivessel disease, poor Killip class on presentation, and was found in ≥ 1 SMuRF group (p-value < 0.01). A higher proportion of patients in the SMuRFless group required emergency PCIs or CABGs.

**Table 2:** Cardiologic data and treatment during the index hospitalization

Variables	Total	SMuRFless	≥ 1 SMuRF	p-value
	N = 15051	N = 2267	N = 12784	
	n (%)	n (%)	n (%)	
**Standard exercise stress test**	275 (17.0%)	29 (14.6%)	246 (17.3%)	0.39
Low*	115 (43.9%)	18 (64.3%)	97 (41.5%)	0.01
Intermediate	43 (16.4%)	5 (17.9%)	38 (16.2%)	
High	99 (37.8%)	5 (17.9%)	94 (40.2%)	
Unavailable	5 (1.9%)	0(0%)	5 (2.1%)	
**Stress echocardiogram**	350 (21.6%)	44 (22.2%)	306 (21.6%)	0.83
Low*	201 (60.4%)	29 (69.0%)	172 (59.1%)	0.25
Intermediate	43 (12.9%)	4 (9.5%)	39 (13.4%)	
High	88 (26.4%)	9 (21.4%)	79 (27.1%)	
Unavailable	1 (0.3%)	0 (0%)	1 (0.3%)	
**Stress testing SPECT MPI**	758 (46.7%)	85 (42.7%)	673 (47.3%)	0.19
Low*	285 (38.3%)	36 (42.4%)	249 (37.7%)	0.97
Intermediate	123 (16.5%)	8 (9.4%)	115 (17.4%)	
High	334 (44.8%)	41 (48.2%)	293 (44.4%)	
Unavailable	3 (0.4%)	0 (0%)	3 (0.5%)	
Cardiac arrhythmia	81 (1.7%)	21 (3.4%)	60 (1.4%)	< 0.01
**LV dysfunction**				0.31
> 50%	484 (62.9%)	48 (56.5%)	436 (63.7%)	
35%-50%	221 (28.7%)	27 (31.8%)	194 (28.4%)	
< 35%	64 (8.3%)	10 (11.8%)	54 (7.9%)	
**Type of ACS**				< 0.01
Unstable angina	1923 (12.8%)	220 (9.7%)	1703 (13.3%)	
NSTEMI	6733 (44.7%)	884 (39.0%)	5849 (45.8%)	
STEMI	6395 (42.5%)	1163 (51.3%)	5232 (40.9%)	
**Killip class**				< 0.01
1	12120 (80.5%)	1942 (85.7%)	10178 (79.6%)	
2	1297 (8.6%)	149 (6.6%)	1148 (9.0%)	
3	1247 (8.3%)	122 (5.4%)	1125 (8.8%)	
4	387 (2.6%)	54 (2.4%)	333 (2.6%)	
Heart failure at presentation	1826 (12.1%)	189 (8.3%)	1637 (12.8%)	< 0.01
**Diagnostic cath**	12487 (97.2%)	1855 (97.1%)	10632 (97.2%)	0.78
Elective	1307 (10.5%)	142 (7.7%)	1165 (11.0%)	
Urgent	7265 (58.2%)	1027 (55.4%)	6238 (58.7%)	
Emergency	3796 (38.4%)	664 (35.8%)	3132 (29.4%)	
Salvage	112 (0.9%)	20 (0.2%)	92 (0.9%)	
**Number of vessels with coronary artery disease**				< 0.01
None	1315 (10.3%)	230 (12.0%)	1085 (9.9%)	
Single	5140 (40.1%)	894 (46.7%)	4246 (38.9%)	
Double	3164 (24.7%)	458 (24.0%)	2706 (24.8%)	
Triple	3209 (25.0%)	329 (17.2%)	2880 (26.4%)	
**PCI**	7056 (46.9%)	1201 (53.0%)	5855 (45.8%)	< 0.01
Elective*	396 (5.6%)	43 (3.6%)	353 (6.0%)	< 0.01
Urgent	3413 (48.4%)	532 (44.4%)	2881 (49.1%)	
Emergency	3228 (45.7%)	618 (51.5%)	2610 (44.5%)	
Salvage	25 (0.4%)	6 (0.1%)	19 (0.3%)	
**CABG**	1674 (11.0%)	191 (8.4%)	1483 (11.6%)	< 0.01
Elective*	49 (2.9%)	4 (2.1%)	45 (3.0%)	0.32
Urgent	1519 (90.7%)	172 (90.1%)	1347 (90.8%)	
Emergency	94 (5.6%)	14 (7.3%)	80 (5.4%)	
Salvage	12 (0.7%)	1 (0.5%)	11 (0.7%)	

*Analyzed using linear-by-linear association

*ACS = Acute coronary syndrome*

*PCI = Percutaneous coronary intervention*

*CABG = Coronary artery bypass grafting*

*SMuRF = Standard Modifiable Risk Factor*

*LV = left ventricular*

Table: 3 shows drugs prescribed at the time of discharge. Medical management differed in two groups with a lower proportion of SMuRFless patients prescribed ARB/ACE inhibitors, statins, and aspirin at discharge compared to the other group.

**Table 3:** Medical treatments at discharge

Variables	Total	SMuRFless	≥ 1 SMuRF	p-value
	N = 15051	N = 2267	N = 12784	
	n (%)	n (%)	n (%)	
**Beta-blockers**	12561 (85.4%)	1862 (84.1%)	10699 (85.6%)	0.06
**Statins**	13387 (88.9%)	1957 (86.3%)	11430 (89.4%)	< 0.01
**Aspirin**	13402 (89.0%)	1964 (86.6%)	11438 (89.7%)	< 0.01
**ACE-I/ARB**	10447 (69.4%)	1540 (68.1%)	8907 (69.8%)	0.1

*ACE-I/ARB = Angiotensin converting enzymes/Angiotensin Receptor Blocking agent*

*SMuRF = Standard Modifiable Risk Factor*

### In-hospital mortality

There was no difference in the in-hospital mortality or complications between the groups. Table: 4 shows that in-hospital mortality was 4.1% in SMuRFless and 3.9% in the ≥ 1 SMuRF group.

**Table 4:** In-hospital outcomes of the patients

Variables	Total	SMuRFless	≥ 1 SMuRF	p-value
	N = 15051	N = 2267	N = 12784	
	n (%)	n (%)	n (%)	
In-hospital death	590 (3.9%)	93 (4.1%)	497 (3.9%)	0.59
Reinfarction	40 (0.3%)	8 (0.4%)	32 (0.3%)	0.38
Cardiogenic shock	259 (1.7%)	41 (1.8%)	218 (1.7%)	0.72
Heart failure	140 (0.9%)	19 (0.8%)	121 (0.9%)	0.62
Stroke*	23 (0.2%)	7 (0.3%)	16 (0.1%)	0.07
New requirement for dialysis	62 (0.4%)	11 (0.5%)	51 (0.4%)	0.55
Gastrointestinal bleeding*	16 (0.1%)	1 (0.0004%)	15 (0.1%)	0.49
Genitourinary bleeding*	10 (0.1%)	2 (0.1%)	8 (0.1%)	0.65
Stent thrombosis*	16 (0.1%)	5 (0.002%)	11 (0.0001%)	0.08

*Fischer exact test was applied

*SMuRF = Standard Modifiable Risk Factor*

The adjusted model of in-hospital mortality is depicted in Table: 5. SMuRFless was not associated with in-hospital mortality; AOR 1.1 (95% CI 0.8, 1.3). Age > 50 years and women had higher odds of in-hospital mortality compared to the patients who were less than or equal to 50 years of age and male patients. There was a graded increase in in-hospital mortality among those presenting with NSTEMI and STEMI compared with those presenting with unstable angina, and with worsening of Killip class at presentation. An increase in the number of diseased vessels also had increased odds of mortality that ranged from 1.8 times higher in single vessel disease to 3.5 times higher in triple vessel disease. CABG also increased the odds twice compared to those who did not undergo CABG.

**Table 5:** Multivariable model of associated factors of in-hospital mortality

Variables	Crude Odds ratio	Adjusted odds ratio
	(95% CI)	(95% CI)
SMuRFless	1.0 (0.8, 1.3)	1.1 (0.8, 1.3)
≥ 1 SMuRF	1	1
**Age**		
≤ 50	1	1
> 50	2.4 (1.9, 3.1)	1.7 (1.3, 2.3)
Women	1.5 (1.2, 1.8)	1.4 (1.1, 1.8)
Men	1	1
**Type of ACS**		
Unstable angina	1	1
NSTEMI	3.1 (1.8, 5.2)	1.4 (0.7, 2.8)
STEMI	8.8 (5.2, 14.8)	4.5 (2.3, 9.0)
**Killip class**		
1	1	1
2	4.8 (3.7, 6.1)	3.3 (2.4, 4.4)
3	7.0 (5.6, 8.9)	5.7 (4.4, 7.5)
4	49.6 (38.8, 63.5)	24.7 (18.6, 32.7)
**Number of vessels with coronary artery disease**		
None	1	1
Single	2.7 (1.5, 4.8)	1.8 (1.0, 3.4)
Double	4.1 (2.3, 7.3)	2.4 (1.3, 4.5)
Triple	6.6 (3.8, 11.7)	3.5 (1.9, 6.2)
**PCI**		
Yes	1.1 (0.9, 1.3)	1.1 (0.9, 1.5)
No	1	1
**CABG**		
Yes	1.5 (1.2, 1.9)	2.0 (1.4, 2.6)
No	1	1

*ACS = Acute Coronary Syndrome*

*PCI = Percutaneous coronary intervention*

*CABG = coronary artery bypass grafting*

*SMuRF = Standard Modifiable Risk Factor*

### Sub-group analysis of factors of in-hospital mortality among STEMI and NSTEMI

#### STEMI

In-hospital mortality in STEMI was not different between SMuRFless in ≥ 1 SMuRF group. Table: 6 displayed that the mortality was higher in STEMI patients who were > 50 years of age, women, presented with advanced Killip class, and had multiple diseased vessels.

**Table 6:** Multivariable model of associated factors of in-hospital mortality in STEMI and NSTEMI

Variables	STEMI	NSTEMI
	N = 5927	N = 5879
	Adjusted Odds ratio	Adjusted Odds ratio
	**(95% CI)**	**(95% CI)**
SMuRFless	0.9 (0.6, 1.2)	2.2 (1.3, 3.8)
≥ 1 SMuRF	1	1
**Age**		
≤ 50	1	1
> 50	1.7 (1.2, 2.3)	1.6 (0.8, 3.0)
Women	1.5 (1.1, 1.9)	1.2 (0.8, 1.9)
Men	1	1
**Killip class**		
1	1	1
2	3.3 (2.3, 4.5)	3.6 (2.0, 6.6)
3	6.2 (4.5, 8.7)	5.2 (3.2, 8.3)
4	24.7 (18.1, 33.7)	21.9 (10.4, 45.8)
**Number of vessels with coronary artery disease**		
None	1	1
Single	2.1 (0.9, 4.6)	1.7 (0.6, 4.7)
Double	2.5 (1.1, 5.8)	2.5 (0.9, 6.9)
Triple	4.0 (1.8, 8.8)	2.8 (1.0, 7.4)
**PCI**		
Yes	1.1(0.8, 1.5)	0.9(0.5, 1.6)
No	1	1
**CABG**		
Yes	1.1 (0.7, 1.7)	3.6 (2.2, 5.9)
No	1	1

*PCI = Percutaneous coronary intervention*

*CABG = Coronary artery bypass grafting*

*SMuRF = Standard Modifiable Risk Factor*

#### NSTEMI

Table: 6 showed NSTEMI patients, SMuRFless were twice likely to have in-hospital mortality compared to those with risk factors. The other significant factors of higher odds of in-hospital mortality were Killip class and undergoing CABG.

### 5-year mortality

**Fig. 2:**
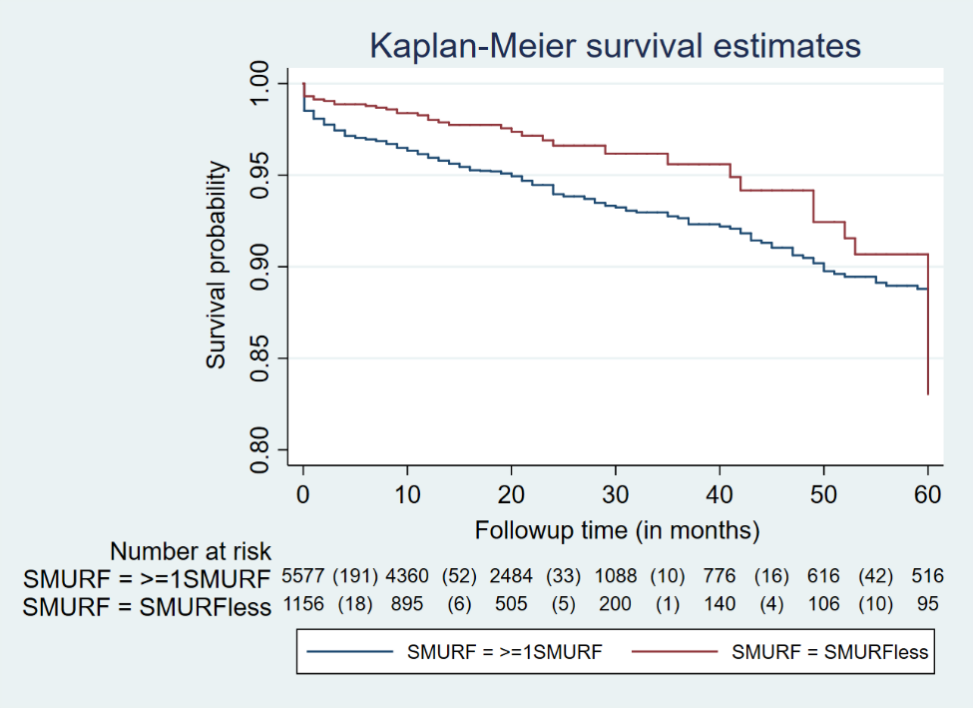
Kaplan Meier curves of survival probability over 60 months for SMuRFless and ≥ 1 SMuRF groups

The follow-up data of 6,733 discharged alive patients (46.6%) was available [SMuRFless: 50.9% (n = 1156); ≥1 SMuRF: 43.6% (n = 5577)]. Total number of deaths on followup was 388(5.76%). Approximately 344(6.17%) deaths in ≥ 1 SMURF group whereas 44(3.81%) in the SMURFless group. The survival probabilities for the two groups are shown in Fig. 2. The Kaplan-Meier graph in Fig. 2 depicts that the survival probability in the SMuRFless group was higher than the ≥ 1 SMuRF group (log-rank test p-value = 0.004). The unadjusted 5-year mortality was 40% lower in the SMuRFless group compared to those who had at least one risk factor (HR: 0.6 95% CI(0.4, 0.8). The multivariable model for 5-year mortality is displayed in Table: 7. The model showed that the 5-year mortality was not different between SMuRFless and ≥ 1 SMuRF groups when age, gender, disease at presentation, its severity, and management were added in the multivariable model (HR:0.7 95% CI(0.5, 1.0). The 5-year mortality hazard was highest for the STEMI cases (2.9 [95% CI 1.3, 6.2]) compared to other ACS presentations. An increase in mortality hazard was also observed with the worsening of the Killip class and the increase in the number of diseased vessels. The hazard was lower if the patient had undergone PCI, or if ACE-I/ARB inhibitors were prescribed at discharge.

**Table 7:** Shows the Multivariable model of associated factors of overall 5-year mortality

Variables	Crude Hazard ratio	Adjusted hazard ratio
N = 6733	(95% CI)	(95% CI)
SMuRFless	0.6 (0.4, 0.8)	0.7 (0.5, 1.0)
≥ 1 SMuRF	1	1
**Age**		
≤ 50	1	1
> 50	3.2 (2.4, 4.6)	2.5 (1.8, 3.4)
Women	1.6 (1.2, 2.0)	1.2 (1.0, 1.6)
Men	1	1
Welfare received for the episode of care	1.4 (0.9, 2.2)	-
**Type of ACS**		
Unstable angina	1	1
NSTEMI	2.8 (1.3, 6.1)	2.5 (1.1, 5.4)
STEMI	2.4 (1.1, 5.3)	2.9 (1.3, 6.2)
**Killip class**		
1	1	1
2	2.3 (1.7, 3.1)	1.8 (1.3, 2.4)
3	4.8 (3.6, 6.3)	3.4 (2.4, 4.5)
4	2.5 (1.4, 4.5)	1.9 (1.0, 3.4)
**Number of vessels with coronary artery disease**		
None	1	1
Single	1.0 (0.7, 1.4)	1.5 (1.0, 2.0)
Double	1.1 (0.7, 1.5)	1.8 (1.2, 2.7)
Triple	1.6 (1.1, 2.3)	1.8 (1.2, 2.6)
**LV dysfunction**		-
> 50%	1	
35%-50%	0.7 (0.1, 3.8)	
< 35%	0.5 (0.1, 5.8)	
**PCI**		
Yes	0.4 (0.3, 0.5)	0.4(0.3, 0.6)
No	1	1
**CABG**		
Yes	1.1 (0.7, 1.5)	-
No	1	
ACE-I/ARB prescribed at discharge	0.5 (0.4, 0.6)	0.6 (0.5, 0.8)
Aspirin prescribed at discharge	0.6 (0.4, 0.9)	0.9 (0.6, 1.6)
Statin prescribed at discharge	0.6 (0.4, 0.9)	0.9 (0.6, 1.4)
Beta-blockers prescribed at discharge	0.7 (0.5, 0.9)	0.9 (0.6, 1.2)

*ACS = Acute Coronary Syndrome*

*LV = left ventricular*

*PCI = Percutaneous coronary intervention*

*CABG = coronary artery bypass grafting*

*ACE-I/ARB = Angiotensin converting enzymes/Angiotensin Receptor Blocking agent*

*SMuRF = Standard Modifiable Risk Factor*

### Sub-group analysis of factors of 5-year mortality among STEMI and NSTEMI

#### STEMI

In multivariable analysis among STEMI patients in Table: 8, being SMuRFless reduced the hazard of 5-year mortality by 40% compared to the ≥ 1 SMuRF group. The prescription of ACE-I/ARB at discharge also had lower hazards of 5-year mortality in STEMI. The factors with an increased hazard of 5-year mortality included age > 50 years, worsening Killip class, and the number of diseased vessels.

**Table 8:** Multivariable model of associated factors of 5-year mortality in STEMI and NSTEMI

Variables	STEMI	NSTEMI
	N = 3379	N = 3113
	Adjusted Hazard ratio	Adjusted hazard ratio
	**(95% CI)**	**(95% CI)**
SMuRFless	0.6 (0.4, 0.9)	0.9 (0.5, 1.5)
≥ 1 SMuRF	1	1
**Age**		
≤ 50	1	1
> 50	2.9 (1.9, 4.5)	2.1 (1.3, 3.4)
Women	1.0 (1.7, 1.5)	1.4 (1.0, 2.0)
Men	1	1
**Killip class**		
1	1	1
2	1.8 (1.2, 2.6)	1.8 (1.1, 2.8)
3	3.0 (1.9, 4.7)	3.6 (2.5, 5.3)
4	2.0 (1.0, 3.8)	0.6 (0.1, 4.9)
**Number of vessels with coronary artery disease**		
None	1	1
Single	1.4 (0.8, 2.3)	1.5 (1.0, 2.4)
Double	1.5 (0.8, 2.7)	2.1 (1.2, 3.5)
Triple	1.9 (1.0, 3.6)	1.5 (0.9, 2.5)
ACE-I/ARB prescribed at discharge	0.6 (0.4, 0.8)	0.7 (0.5, 1.0)
Aspirin prescribed at discharge	0.8 (0.4, 1.7)	1.0 (0.5, 2.0)
Statin prescribed at discharge	0.7 (0.4, 1.3)	1.3 (0.7, 2.5)
Beta-blockers prescribed at discharge	0.9 (0.5, 1.5)	0.9 (0.6, 1.5)

*ACS = Acute Coronary Syndrome*

*ACE-I/ARB = Angiotensin converting enzymes/Angiotensin Receptor Blocking agent*

*SMuRF = Standard Modifiable Risk Factor*

#### NSTEMI

Table: 8 demonstrated that 5-year mortality in NSTEMI was not associated with SMuRF (AHR 0.9, 95% CI 0.5, 1.5). Age > 50 years, presenting with advanced Killip class, and the number of diseased vessels increased the hazards of 5-year mortality. Unlike STEMI, drugs prescribed at discharge were not associated with long-term mortality in NSTEMI patients.

## Discussion

The study found 15% of patients presenting with ACS were without any SMuRF with a higher proportion of males presenting without SMuRF. A higher proportion of SMuRFless patients had cardiac arrhythmia and STEMI at the time of presentation. Subsequently, there were more emergency PCI in this group. There were higher numbers of in-hospital deaths in the SMuRFless group than ≥ 1 SMuRF group but the difference was non-significant. In sub-group analysis, in-hospital mortality was not different between SMuRFless and ≥ 1 SMuRF group in STEMI patients, however, among NSTEMI patients in-hospital mortality was higher in SMuRFless. The adjusted overall 5-year mortality was not different between the two groups. In STEMI patients, the SMuRFless group had a lower hazard of 5-year mortality but the mortality was not different in the NSTEMI cases between SMuRFless and ≥ 1 SMuRF.

The proportion of SMuRFless ACS patients from western parts of the world showed 17% in Sweden [5], 16% in Australia [4], and 23% of patients in England [11]. The limited data from Asia is inconsistent and the proportion of SMuRFless patients ranged from as high as 20.8% of STEMI patients in India [12] to 8.3% of STEMI being SMuRFless in Singapore [6]. We found 18% of STEMI patients to be SMuRFless. Our rates of SMuRFless in STEMI patients were closer to the study from the sub-continent. The lower rates from Singapore compared to Pakistan and India might have been due to the inherently higher risk of coronary disease in South Asians and the difference in health status, the prevalence of traditional risk factors, and the capture of those risk factors [13].

Contrary to other studies, the SMuRFless patients in our study were approximately two years younger than the compared group and co-morbidities like peripheral arterial disease, history of cancer, or valve surgery were also comparable with the patients with ≥ 1 SMuRF [14, 15]. Despite small differences in baseline characteristics of the two groups, more SMuRFless patients presented with STEMI and cardiac arrhythmia, and more patients underwent emergency revascularization compared to patients with ≥ 1 SMuRF. A possible explanation can be the use of medications to manage the traditional risk factors in the ≥ 1 SMuRF group that might have controlled the severity of the ACS.

The in-hospital and 5-year mortality was not different between the SMuRFless and patients with risk factors in our study. Other studies have found higher in-hospital mortality [5, 16] among the SMuRFless group [14]. Our findings are comparable to the study performed in Singapore by Kong et al. who also found no significant difference in in-hospital mortality between patients with no risk factors and patients with risk factors [6]. Other than the Asian population, our study and the study by Kong et al. had similar findings likely because all types of ACS patients were included, unlike other studies that have reported estimates of either STEM or NSTEMI, separately.

It was observed that a higher number of patients underwent PCI in the SMuRFless group in our sample which was not found in other studies [14]. When we adjusted the multivariable model for in-hospital mortality, the type of ACS came out to be a significant factor and STEMI at presentation had a higher risk of mortality. In the sub-group analysis, there was no association between PCI and in-hospital mortality in STEMI and NSTEMI patients.

In our study, the 5-year mortality was less likely in the STEMI SMuRFless patients vs. STEMI patients with risk factors, and no difference in mortality between the two groups among NSTEMI cases. The literature on long-term survival has shown mixed results depending on the type of ACS and the type of population included. Literature has reported higher long-term mortality in SMuRFless patients with STEMI [5] and no difference or lower mortality in the NSTEMI group [16, 17]. Sia et al. reported no difference in adjusted 1-year mortality for either STEMI or NSTEMI group [17]. The current body of literature suggests that studies conducted on the western population show a higher risk of immediate and long-term mortality in STEMI and lower mortality in NSTEMI [4, 5, 16]. On the other hand, studies including the Asian population found no difference in immediate or long-term survival between SMuRF and SMuRFless cases [15, 17, 18]. However, there are few studies from Asia compared to the data from western countries and conclusions should be made cautiously.

The less use of beta-blockers, statins, and ACE-I/ARBARB in the SMuRFless group found in this study was consistent with the literature; however, the long-term mortality was less likely with the use of ACE-I/ARB in our study which is not consistent with the literature. Figtree et al. reported the association of higher mortality in the SMuRFless group with low prescription of ACE-I/ARB and beta-blocker but we did not find an association of beta-blocker prescription with 5-year mortality [5]. The 5-year survival was associated with the disease condition at presentation and regardless of the medications prescribed at discharge, the mortality was higher in patients with multiple vessel disease, STEMI, and advanced Killip class at presentation. Despite the lack of association between 5-year mortality and prescribed medication in our study, physicians should follow the guideline-directed therapy in ACS patients irrespective of the risk factors. The no difference in overall mortality between SMuRFless and ≥ 1SMuRF ACS cases in our study suggests that apparently risk-free patients have a similar risk of adverse outcomes and should be dealt with with the same clinical urgency and rigor.

Our study is one of the largest studies from South Asia on the topic with > 5000 STEMI and NSTEMI patients. The strength of our study is that the classification of the patients in ACS categories was based on discharge diagnosis, the discharge medications were separately recorded from home medications (pre-hospitalization), and medications were administered during hospitalization. Unlike our study, other large studies such as UK-based data of 118, 177 STEMI patients could not differentiate between the drugs prescribed at discharge compared to the drugs given during hospitalization from their registry data [14].

The limitations of the work include this being a single-center study. The cardiology practices are variable across Pakistan and our study does not capture this variation. Hence, the results should be cautiously generalized to the Pakistani population. The lack of difference in the outcomes between SMuRFless and SMuRF groups could have occurred due to the confounders that were not available in our databases such as socioeconomic factors, dietary patterns, and physical activity [19]. The retrospective study design limits the data collection on variables that are not available in the medical records.

Another limitation due to retrospective study design was the high rate of loss to follow-up. We had the follow-up data 6,733 alive at discharge patients (46.5%) which could have effected the result in over or underestimation of 5-year survival rate. Another point to consider is that the 5-year survival was compared between the groups based on their risk status at the time of the index episode and the development of hypertension, diabetes, dyslipidemia, or tobacco use over the long-term follow-up was not taken into account. The change in risk profile could have affected the 5-year outcome in the SMuRFless group. We caution readers to interpret the study results in consideration with the study limitations.

## Conclusion

There were 1 in 7 patients presented with ACS who were SMuRFless. A higher proportion SMuRFless patients were men and a higher proportion of SMuRFless presented with STEMI and cardiac arrhythmia. The in-hospital and 5-year mortality was not different between the SMuRFless and patients with ≥ 1 SMuRF in the studied ACS cohort. Multicenter studies that include both public and private health facilities in Pakistan with active follow-up are needed to encompass the entire socioeconomic spectrum and differences in the health care providers in the country.

## Data Availability

The datasets used and/or analysed during the current study are available from the corresponding author on reasonable request.

## List of abbreviations

ACS: Acute Coronary Syndrome
CABG: Coronary Artery Bypass Grafting
CHD: Coronary Heart Disease
CI: Confidence Interval
EMR: Electronic Medical Record
HR: Hazard Ratio
NCDR®: National Cardiovascular Data Registry
NSTEMI: Non-ST elevation Myocardial Infarction
OR: Odds Ratio
PCI: Percutaneous Intervention
SA: South Asia
SMuRF: Standard Modifiable Risk Factor
STEMI: ST elevation Myocardial Infarction
THI: Tabba Heart Institute
